# Diagnostic value of *RAS* mutations in Bethesda category IV thyroid nodules: a systematic review and meta-analysis

**DOI:** 10.3389/fonc.2025.1685864

**Published:** 2025-12-10

**Authors:** Jiayue Sun, Yilin Hou, Chengfei Sun, Di Wu, Yunfei Zhang

**Affiliations:** 1 Department of Ultrasound, The First Affiliated Hospital of China Medical University, Shenyang, Liaoning, China; 2 Department of Ultrasound, General Hospital of Fushun Mining Bureau of Liaoning Health Industry Group, Fushun, Liaoning, China; 3 Department of Ultrasound, Liaoning Cancer Institute and Hospital, Shenyang, Liaoning, China

**Keywords:** thyroid nodules, Bethesda category IV, *RAS* mutation, meta analysis, cancer

## Abstract

**Objectives:**

Thyroid nodules classified as Bethesda category IV cannot be diagnosed with fine-needle aspiration. *RAS* mutations have been linked to thyroid cancer, although it is still unclear if they are useful as a pre-operative molecular screening for this particular subgroup. In order to assess the diagnostic accuracy (sensitivity, specificity, AUC, etc.) of *RAS* mutations in identifying malignancy among Bethesda IV nodules and to ascertain whether this can consistently direct clinical decision-making and minimize non-essential interventions, we carried out a systematic review and meta-analysis using postoperative histopathology as the reference standard.

**Methods:**

A literature search of the PubMed, Embase, Cochrane Library, Web of Science and OVID databases was conducted. In all of the included studies, the diagnostic accuracy of *RAS* was compared with that of postoperative pathology, which was used as a standard. Data were pooled, and the sensitivity, specificity, area under the curve (AUC), positive likelihood ratio (PLR), negative likelihood ratio (NLR), and diagnostic odds ratio (DOR) were calculated to estimate the accuracy of *RAS.*

**Results:**

A total of 10 studies were included after screening, comprising 913 cases of Bethesda Category IV nodules. Among them, there were 246 malignant lesions and 667 benign lesions. The pooled sensitivity of *RAS* mutations was 0.35 (95% CI, 0.29-0.41), and the specificity was 0.93 (95% CI, 0.90-0.95). The positive likelihood ratio was 3.46 (95% CI, 1.94-6.18), the negative likelihood ratio was 0.73 (95% CI, 0.60-0.89), and the AUC was 0.7954. Heterogeneity testing showed p<0.05, I^2^ = 58.5%, indicating significant heterogeneity.

**Conclusion:**

*RAS* mutations have a moderate diagnostic value in the diagnosis of Bethesda Category IV thyroid nodules. Despite the presence of some heterogeneity, the detection of *RAS* mutations may be helpful in guiding clinical decision-making and management strategies.

**Systematic Review Registration:**

https://www.crd.york.ac.uk/prospero, identifier CRD42024608197.

## 1 Introduction

Thyroid nodules (TNs) occur in 20–70% of the global population (1). Thyroid nodule diagnosis and treatment have changed dramatically with the introduction of sophisticated diagnostic tools like ultrasound and fine-needle aspiration (FNA). As the gold standard for categorizing and reporting FNA results, the Bethesda System for Reporting Thyroid Cytopathology offers a consistent framework for clinical decision-making. Even with these developments, it is still challenging to classify a portion of thyroid nodules, which leads in imprecise FNA findings. To effectively stratify the risk of malignancy in these instances, especially those categorized as Bethesda III and IV, further diagnostic methods are needed (2). Molecular testing has become a valuable auxiliary means (3), among which *RAS* gene testing has high value in the diagnosis of uncertain thyroid nodules. By improving the diagnostic accuracy, guiding clinical management, risk stratification, reducing unnecessary surgery and evaluating prognosis, it has greatly improved the clinical management of these nodules. In conclusion, this meta-analysis aimed to evaluate the role of *RAS* gene mutations in classifying Bethesda class IV thyroid nodules as benign or malignant. When Bethesda IV nodules are tested negative for *RAS* and have no high-risk imaging or clinical features, most hospitals now choose observation rather than timely surgery to prevent unnecessary lobectomy while maintaining safety (4, 5).

## 2 Materials and methods

### 2.1 Literature search

The study complied with the PRISMA recommendations (6, 7). An independent search of the English medical literature was conducted using the PubMed (Medicine) database, Embase, Cochrane Library, Web of Science, and OVID, to identify all studies involving diagnostic tests that estimated the value of *RAS* for the diagnosis of Bethesda Category IV Thyroid Nodules. Searches were performed using the following keywords: “ras”, “fine needle aspiration”, “Bethesda IV”, “Bethesda 4”, “thyroid”. Duplicate articles were manually excluded. Unpublished relevant data were also considered; however, no studies with such data were found that were appropriate for inclusion.

### 2.2 Eligibility and exclusion criteria

A study was included if it met the following criteria (1): the study assessed the capability of *RAS* in detecting Bethesda Category IV Thyroid Nodules (2); postoperative pathology was used as a diagnostic standard (3); reported data (sensitivity and specificity) necessary to calculate the true-positive (TP), false-negative (FN), false-positive (FP), and true-negative (TN) rates of *RAS* in the diagnosis of Bethesda Category IV Thyroid Nodules. Here, true-positive (TP) denotes a *RAS* mutation detected in pre-operative FNA with post-operative confirmation of malignancy (e.g., follicular carcinoma); false-negative (FN) indicates no *RAS* mutation in FNA but malignancy on histology; false-positive (FP) signifies *RAS* mutation present in FNA yet benign pathology; and true-negative (TN) refers to absence of *RAS* mutation in FNA coupled with benign surgical findings. However, since not all of the included studies performed comprehensive testing for *BRAF, TERT, RET*/*PTC*, and other common driver mutations, a nodule that is *RAS*-negative yet histologically malignant could still harbor an undetected alteration responsible for its malignant behavior. Additionally, in this meta-analysis, non-invasive follicular thyroid neoplasm with papillary-like nuclear features (NIFTP) was classified as malignant. All included studies should have obtained informed consent from study participants and received protocol approval by an ethics committee or institutional review board. Exclusion criteria included (1): data are insufficient (2); review articles, meta-analyses, conference proceedings, reports, letters, editorial comments, opinions, prefaces (3); no postoperative pathology (4); non-English language literature. We did not include in this study any other Bethesda cytology findings except for category IV. All disagreements were resolved by consensus.

### 2.3 Data extraction

All relevant data from the 10 included studies, such as the first author, year of study, country of study, number of lesions, type of lesions, reference standards, and the numbers of true positives, false positives, false negatives, and true negatives, were extracted in a unified format. Any divergence from this procedure was resolved by discussion.

### 2.4 Assessments of methodological quality

Using the QUADAS-2 tool for diagnostic test quality assessment within RevMan 5.4 software, the quality of included literature was evaluated, which entailed assessing the risk of bias and applicability of each included article. The QUADAS-2 tool classifies risks for bias into four key domains: patient selection, index test, reference standard, and flow and timing. Each domain was assessed in terms of the risk of bias, and patient selection, index test, and reference standard were also evaluated for applicability. In the event of any disagreement, it was resolved through negotiation.

### 2.5 Statistical analysis

The statistical software Meta-Disc (Version 1.4, Unit of Clinical Biostatistics team of the Ramón y Cajal Hospital), STATA (Version 18.0, Stata Corporation) and RevMan 5.4 were used in this study. The Spearman correlation coefficient was used to analyze the threshold effect. The heterogeneity was evaluated by the Cochran Q statistic and the I^2^ test. When the p-value for heterogeneity was less than 0.05 or I² was at least 50%, a random effects model was employed; otherwise, a fixed effects model was used. Additionally, meta-regression analysis, subgroup analysis, or sensitivity analysis were conducted to identify the sources of heterogeneity. The pooled sensitivity, specificity, diagnostic odds ratio (DOR), area under the curve (AUC) and Q* index were calculated using Meta-Disc, and a forest plot was generated. Potential sources of heterogeneity were explored with a meta-regression analysis. Deeks’ funnel plot was generated in STATA to analyze the potential publication bias, with a p < 0.05 indicating potential publication bias. During the screening of articles and the application of the QUADAS-2 criteria, the RevMan 5.4 software was utilized to perform analyses that assessed the interobserver agreement.

## 3 Results

### 3.1 Literature search and characteristics of included studies

**Figure 1:**
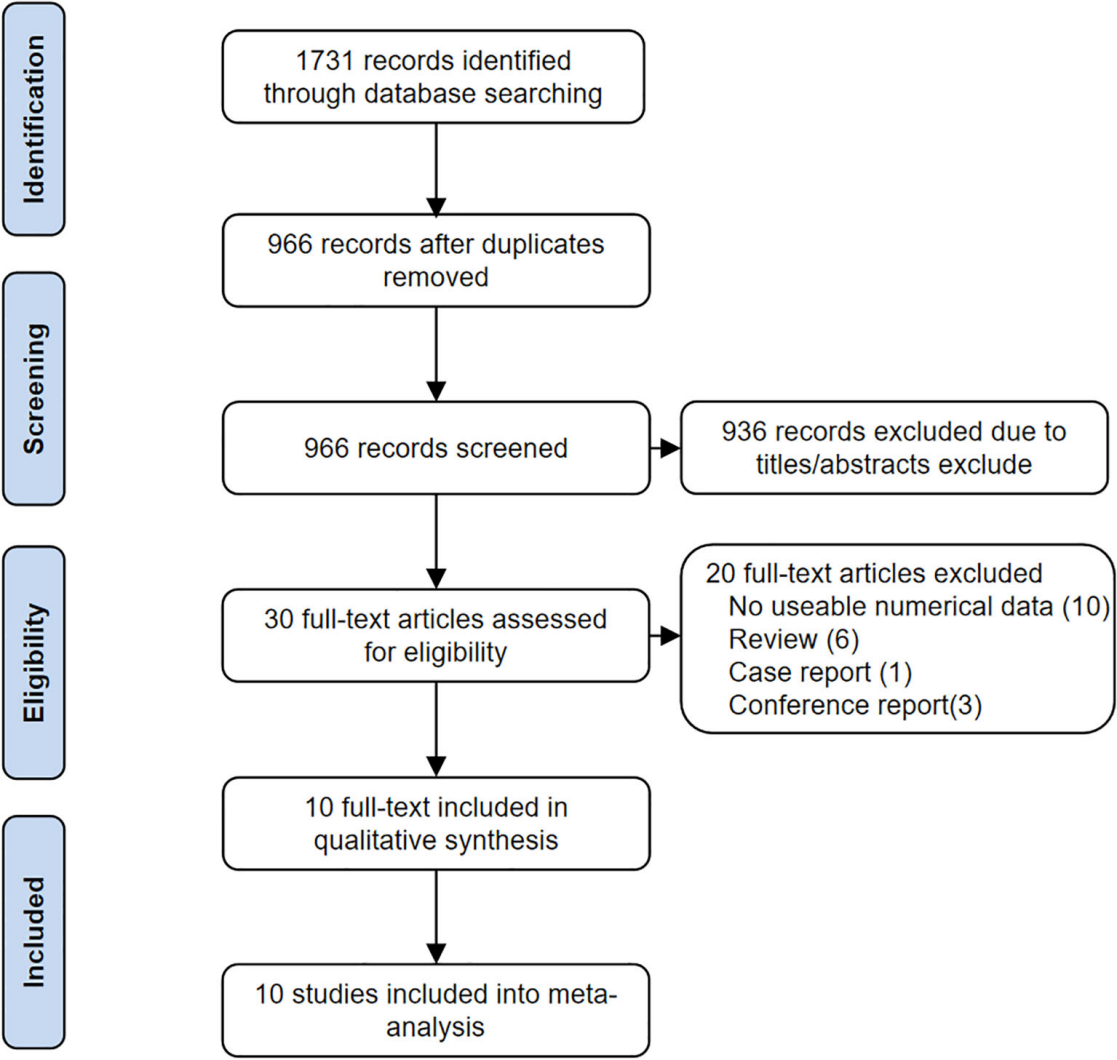
Flow diagram of the study selection process following the preferred reporting items for systematic reviews and meta-analyses (PRISMA) guidelines.

A total of 1731 articles were retrieved. After removing duplicates, 30 articles were preliminarily included by reading the titles and abstracts. After excluding 20 articles with incomplete data and those that did not meet the inclusion criteria, 10 articles were ultimately included. The specific screening process is shown in **Figure 1**. The main characteristics of the included studies are summarized in **Table 1**.

**Table 1:** Main characteristics of included studies.

No.	Author	Country	Year	Type	Number of lesions	TP	FP	FN	TN
1	Yuri E. (8)	USA	2011	prospective study	210	29	5	25	151
2	Sylvie Beaudenon-Huibregtse (24)	USA	2014	prospective study	19	4	1	2	12
3	Aaron A. Stence (25)	USA	2015	retrospective study	18	3	4	4	7
4	Luigi De Napoli (11)	Italy	2016	retrospective study	258	22	9	68	159
5	Rupendra T. Shrestha (26)	USA	2016	retrospective study	12	5	3	0	4
6	Hongxun Wu (20)	China	2019	retrospective study	23	3	2	5	13
7	Jee Hyun An (16)	Japan	2015	prospective study	8	2	0	4	2
8	Markus Eszlinger (27)	Canada	2017	prospective study	181	5	13	29	134
9	M. Decaussin-Petrucci (21)	France	2017	prospective study	124	3	9	13	99
10	Yoon Young Cho (22)	Korea	2020	prospective study	60	9	3	11	36
No.	Type of lesion			Reference standard		SE(%)		SP(%)	
1	RAS(+):21 PTC,FV, 5 PTC, 3 FTC,5 FA RAS (–):16 PTC,FV, 3 PTC, 6 FTC,95 HN, 56 FA			postoperative pathology		54		97	
2	NA			postoperative pathology		67		92	
3	RAS(+):1PTC, CV,2FVPTC,2FA,1Nodular hyperplasia,1Adenomatoid nodule RAS (–):4FA,1FC,2FVPTC,2PTC,1Benign nodule,1Adenomatoid nodule			postoperative pathology		43		64	
4	NA			postoperative pathology		24		95	
5	NA			postoperative pathology		100		57	
6	NA			postoperative pathology		38		87	
7	RAS (–)and benign:1FA 1NH RAS(+)and malignancy:1FVPTV 1PTC			postoperative pathology		33		100	
8	RAS(+):1AN,9FA,3OFA,1FTC,3FVPTC,1CPTC RAS (–):3Thyroiditis,39AN,59FA,33OFA,7FTC,6OFTC,9FVPTC,7CPTC			postoperative pathology		15		91	
9	NA			postoperative pathology		19		92	
10	RAS(+):6 FVPTC (3 NIFTP), 2 FTC, 1Warthin-like variant of PTC, 3 FA RAS (–):5 FVPTC (1 NIFTP), 3 FTC, 3 oncocytic variant of FTC, 22 FA, 6 oncocytic FA, 8 HN			postoperative pathology		45		92	

### 3.2 Data extraction and quality assessment of the literature

**Figure 2:**
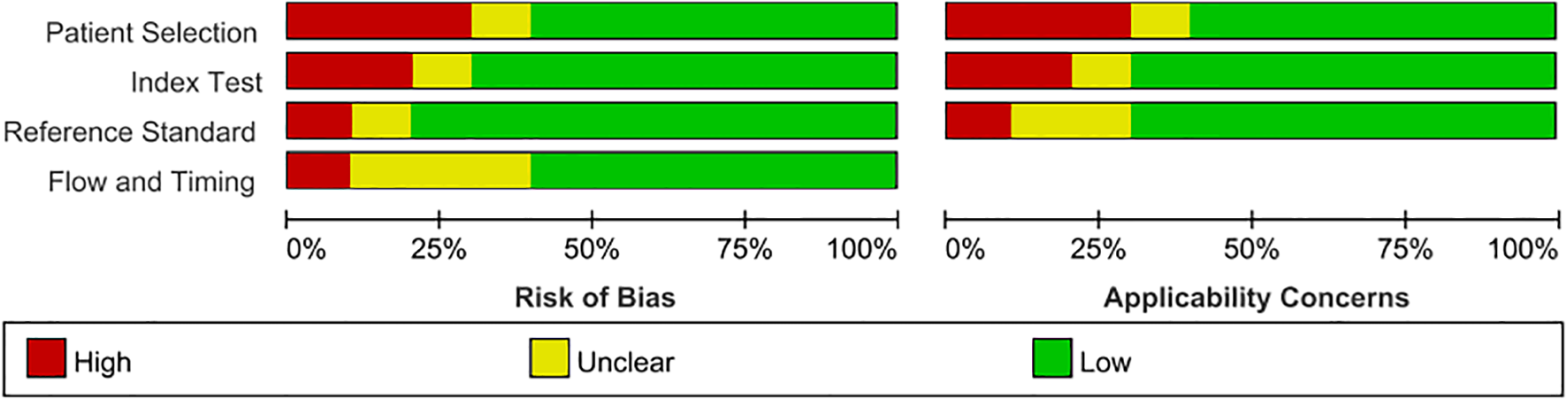
Risk of bias and applicability concerns graph. The horizontal axis shows the percentage (0–100%) of studies assigned to each risk level (green = low, red = high, yellow = unclear); the vertical axis lists the four QUADAS-2 domains. The left panel summarizes the overall risk of bias, and the right panel summarizes applicability concerns, indicating how well the findings can be applied to our research question. The overall risk of bias is low, but some studies have unclear bias in the index test.

**Figure 3:**
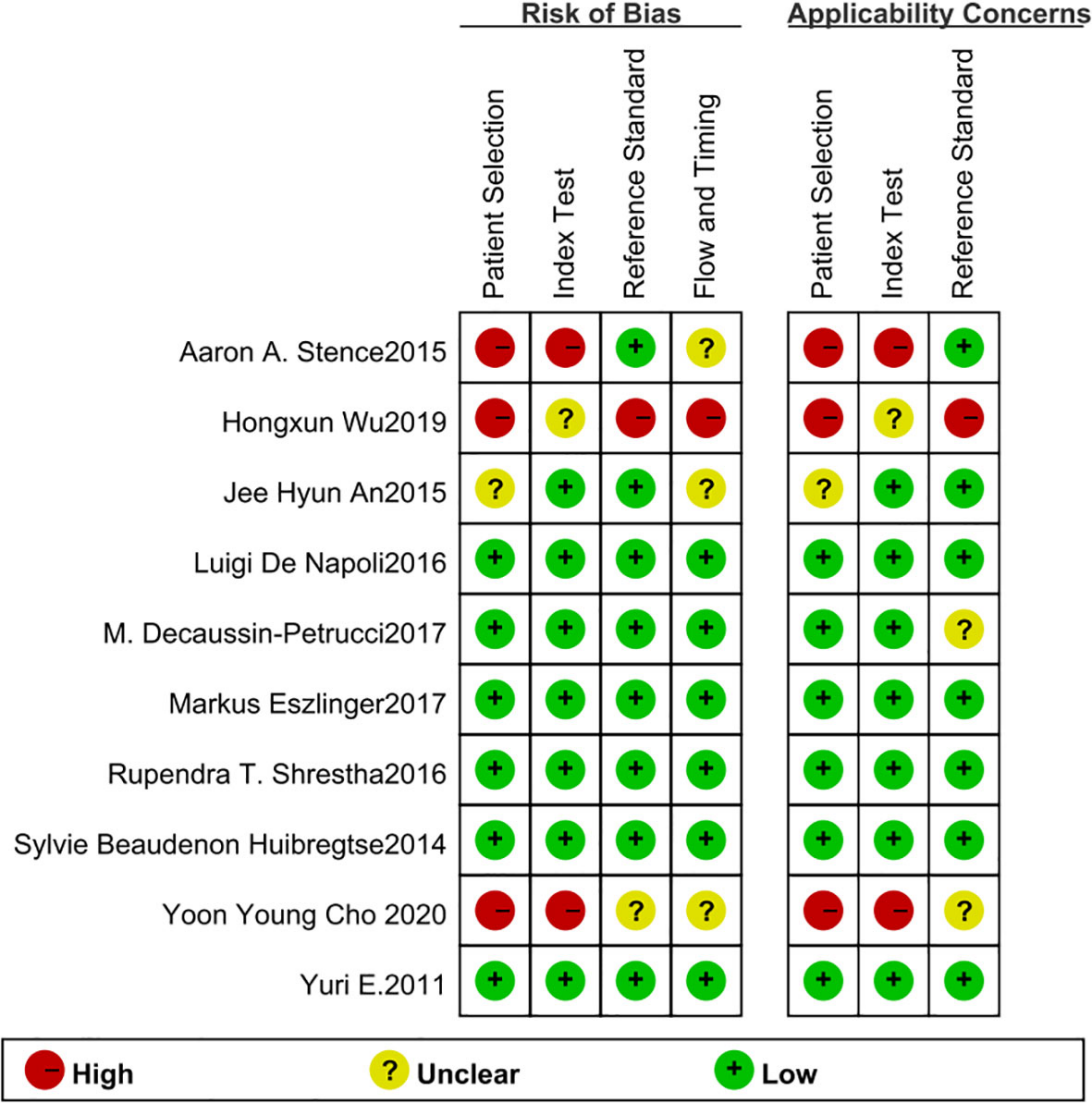
Risk of bias and applicability concerns summary. The horizontal axis presents the four QUADAS-2 core domains (Patient Selection, Index Test, Reference Standard, Flow and Timing), while the vertical axis lists each included study. The left four columns show the risk-of-bias judgments for every domain, and the right three columns summarize whether the results are applicable to our research question. The predominance of green “+” symbols indicates an overall low risk of bias across the included studies.

The included 10 articles comprised 4 retrospective studies and 6 prospective studies, encompassing a total of 913 lesions, of which 246 were malignant and 667 were benign, with a positive rate of 26.94% and a negative rate of 73.06%. The QUADAS-2 results indicated that the overall risk of bias across the studies was low, with quality assessment results shown in **Figures 2**, **3**.

### 3.3 Diagnostic accuracy for Bethesda category IV thyroid nodules

**Figure 4:**
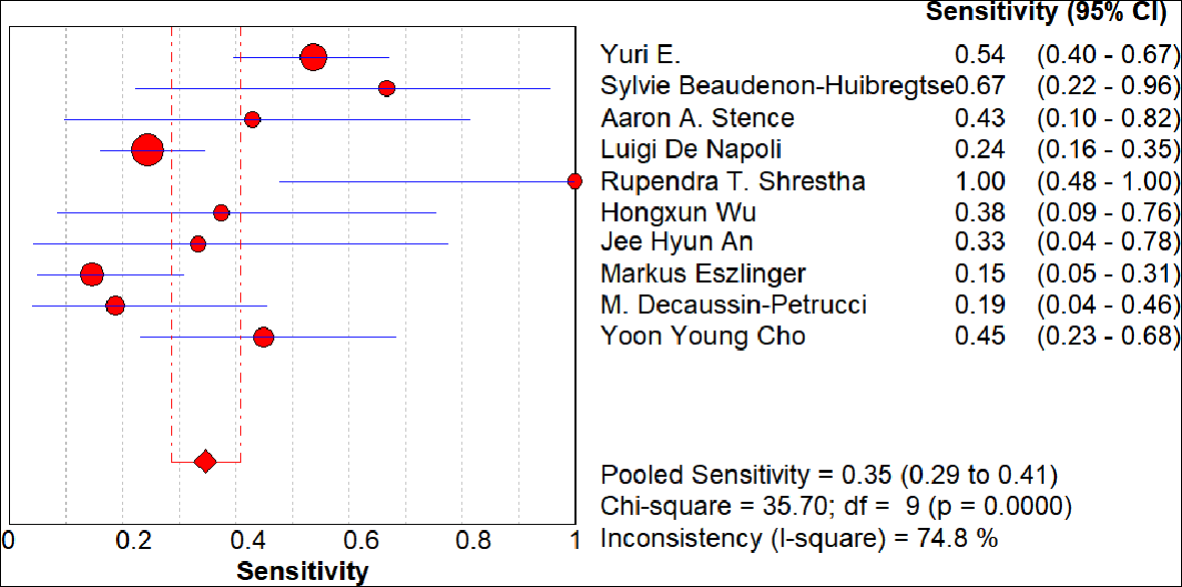
Forest plots of the pooled sensitivity. Each circle represents the point estimate of sensitivity for an individual study, with the circle size proportional to the sample size; the horizontal line denotes the 95% confidence interval (CI), where a shorter line indicates higher precision. The diamond at the bottom shows the pooled sensitivity from the random-effects model (0.35; 95% CI: 0.29–0.41), and the width of the diamond reflects the precision of the pooled estimate. An I² of 74.8% and p < 0.01 indicate substantial heterogeneity among studies.

**Figure 5:**
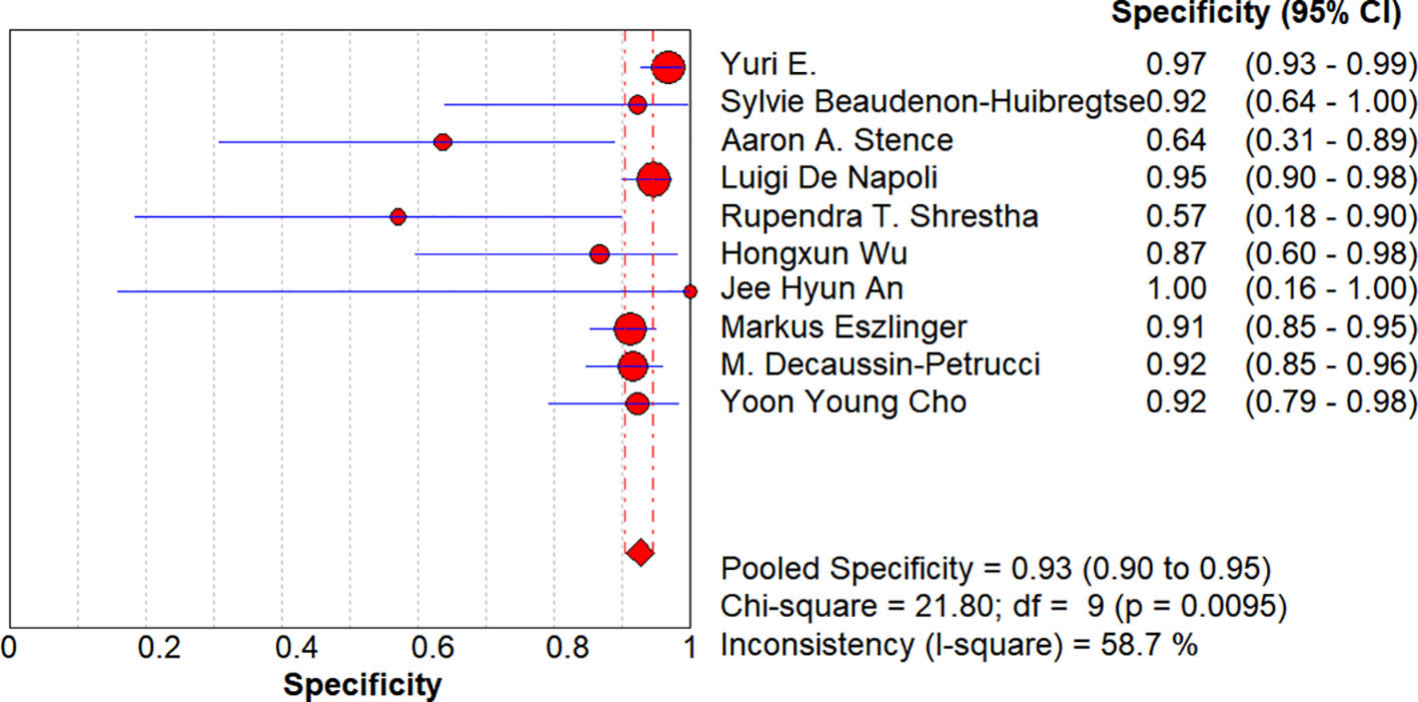
Forest plots of the pooled specificity. Each circle represents the study-specific point estimate of specificity; the circle size is proportional to the sample size and the number of true-negative events. The horizontal line denotes the 95% CI, with shorter lines indicating higher precision. The diamond at the bottom shows the random-effects pooled specificity (0.93; 95% CI: 0.90–0.95). I² = 58.7% indicates moderate heterogeneity.

**Figure 6:**
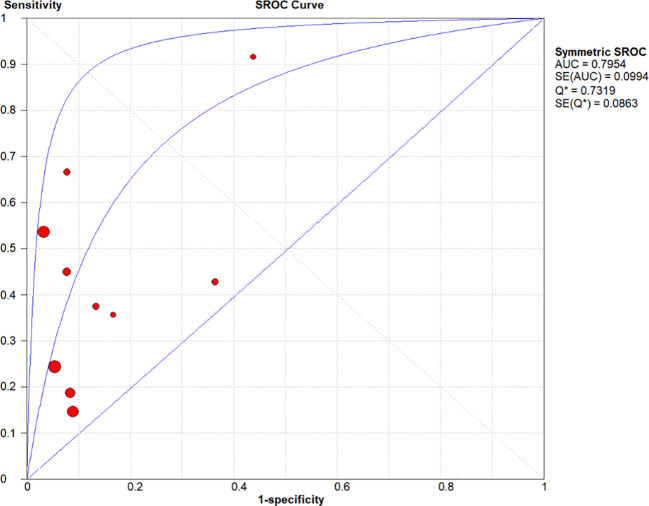
Summary receiver operating characteristic (SROC) curve. Horizontal axis (X): 1 − Specificity (false-positive rate); moving from 0 to 1 indicates increasing false-positive frequency—positions farther left denote fewer misdiagnoses and higher specificity. Vertical axis (Y): Sensitivity (true-positive rate); moving from 0 to 1 indicates increasing detection—positions farther up denote fewer missed cases and higher sensitivity. The central blue symmetric curve is the fitted summary ROC, representing the pooled diagnostic performance across all studies; the closer the curve is to the top-left corner, the higher the overall accuracy. The circular dots are the actual coordinate points of the included studies; they scatter around the summary ROC curve, illustrating the degree of deviation of individual studies from the pooled estimate. An AUC of 0.7954 indicates moderate-to-good diagnostic accuracy, and a Q* value of 0.7319 supports this interpretation.

**Figure 7:**
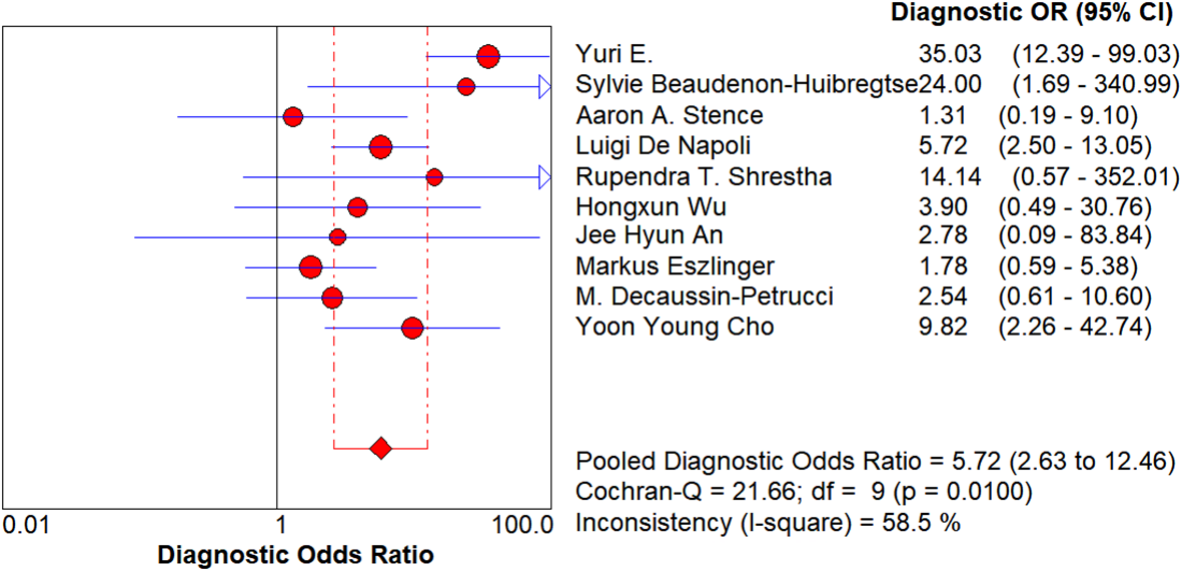
Forest plot of diagnostic odds ratio. Each circle represents the study-specific diagnostic odds ratio (DOR) point estimate; the circle size is proportional to both the sample size and the number of events. The horizontal line denotes the 95% confidence interval (CI), with shorter lines indicating higher precision. The diamond at the bottom shows the random-effects pooled DOR (5.72; 95% CI: 2.63–12.46). I² = 58.5% suggests moderate heterogeneity.

**Figure 8:**
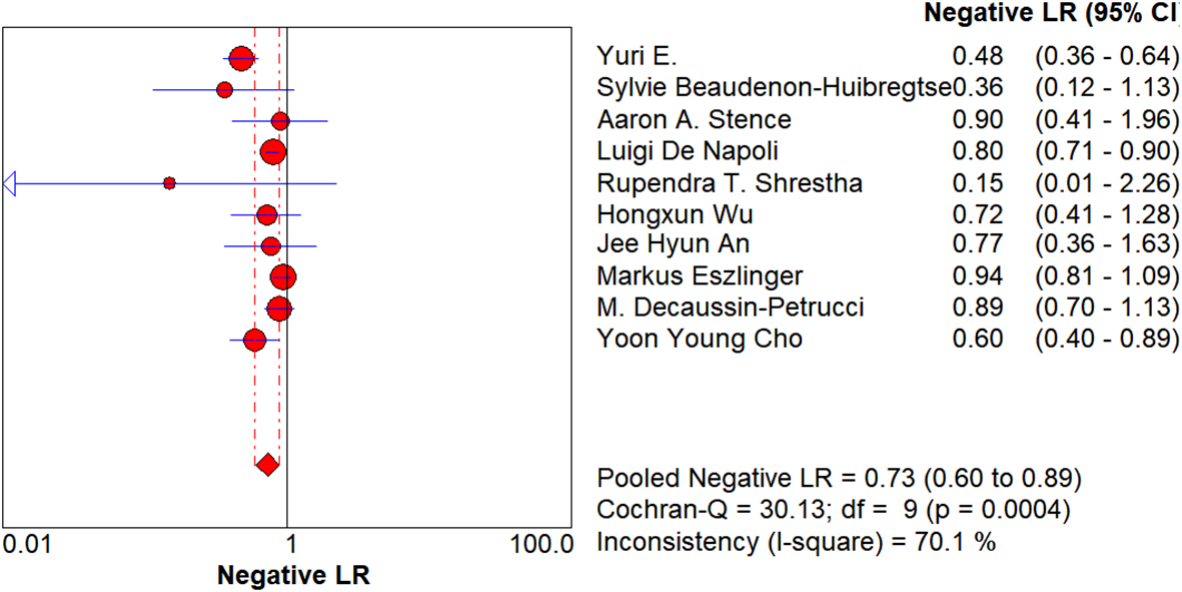
Forest plot of negative likelihood ratio. Each circle represents the study-specific negative likelihood ratio (Negative LR) point estimate; the circle size is proportional to both the sample size and the number of events. The horizontal line denotes the 95% confidence interval (CI), with shorter lines indicating higher precision. The diamond at the bottom shows the random-effects pooled Negative LR (0.73; 95% CI: 0.60–0.89). I² = 70.1% indicates moderate-to-high heterogeneity. RAS negative only reduced the malignant probability to approximately 70%, and the exclusion power was weak; if there are no high-risk imaging/clinical signs, conservative follow-up is advisable.

**Figure 9:**
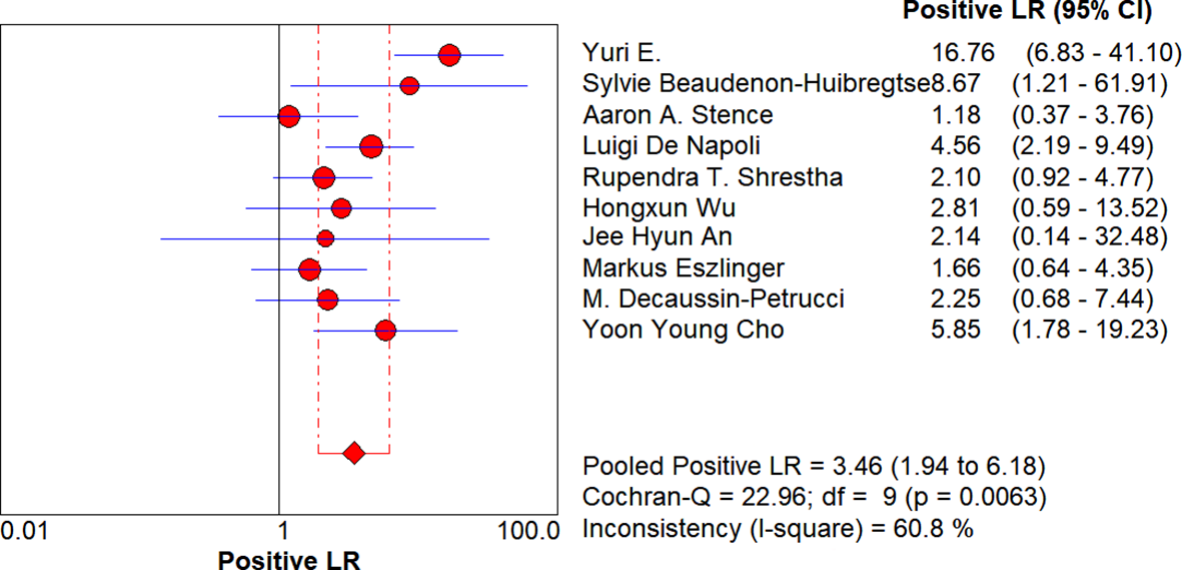
Forest plot of positive likelihood ratio. Each circle represents the study-specific positive likelihood ratio (Positive LR) point estimate; the circle size is proportional to both the sample size and the number of events. The horizontal line denotes the 95% confidence interval (CI), with shorter lines indicating higher precision. The diamond at the bottom shows the random-effects pooled Positive LR (3.46; 95% CI: 1.94–6.18). I² = 60.8% indicates moderate heterogeneity. When RAS is positive, the malignant probability increases approximately 3.5-fold, and surgery is usually considered.

The summary receiver operating characteristic(SROC) curve of the included 10 articles showed a distribution that was not “shoulder-arm” shaped, and no threshold effect was identified by analysis of the diagnostic threshold, with a Spearman correlation coefficient of 0.055(p = 0.881). The Cochran Q test and the I^2^ test revealed significant heterogeneity with p < 0.05 and I^2^ = 58.5%. Based on the results of the heterogeneity assessment, a random-effects model was used for the meta-analysis. The diagnostic accuracy of *RAS* for Bethesda Category IV thyroid nodules was computed on the basis of a pooled sensitivity of 0.35 (95% CI 0.29–0.41), specificity of 0.93 (95% CI 0.90– 0.95), positive LR of 3.46 (95% CI 1.94–6.18), negative LR of 0.73 (95% CI 0.60–0.89) and DOR of 5.72 (95% CI 2.62–12.46). An overall moderate degree of accuracy was identified by the SROC curve with an AUC of 0.80 (Q* = 0.73) (**Figures 4**–**9**). Some studies also tested for mutations other than *RAS*. In those reports, several cancers that were *RAS*-negative turned out to carry *BRAF, TERT* or *RET*/*PTC* changes, showing that they could have been flagged by a broader gene panel. Therefore the number of “FN” we recorded over-estimates the real shortcoming of *RAS* testing alone.

### 3.4 Heterogeneity results

To explore the potential sources of heterogeneity not caused by threshold effects, univariate Meta regression analysis was employed, successively examining variables such as publication year (before and after 2016), detection of *RAS* typing (all detected/only 1–2 types detected), study type (prospective study/retrospective study), number of lesions (median 42; more than 42/less than or equal to 42), country (Asia and non-Asia), and research center (multicenter/single center), but no clear source of heterogeneity was identified.

### 3.5 Sensitivity analysis

**Figure 10:**
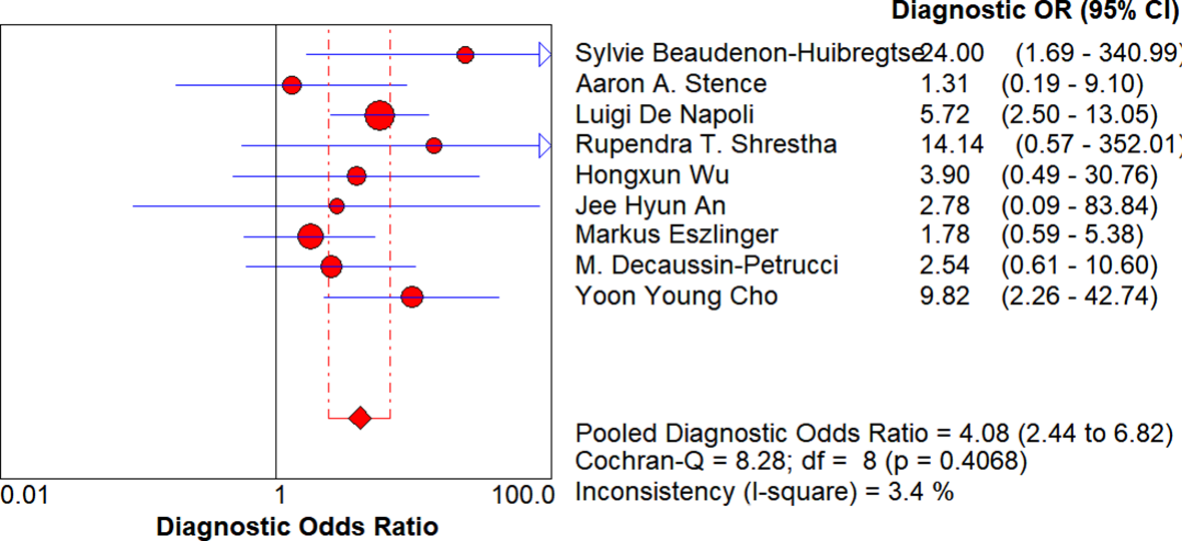
Forest plot of diagnostic odds ratio(After removing Yuri E.). After excluding this study, I² dropped from 58.5% to 3.4%, indicating it was the main driver of heterogeneity and a key influence on the pooled diagnostic performance.

A sensitivity analysis was carried out by eliminating each study individually in order to better investigate the stability and sources of heterogeneity in this investigation. The confidence interval of Yuri E. et al. (8) considerably reduced overlap with the confidence intervals of other research, according to the sensitivity analysis forest plot, suggesting that this study may demonstrate heterogeneity with other studies. After excluding Yuri E. et al. (8), Q = 8.28, df=8, p=0.4068 (p>0.05), and the I^2^ value decreased from 58.5% to 3.4% (**Figure 10**), further indicating that Yuri E. et al. (8) was the source of heterogeneity.

### 3.6 Evaluation of publication bias

**Figure 11:**
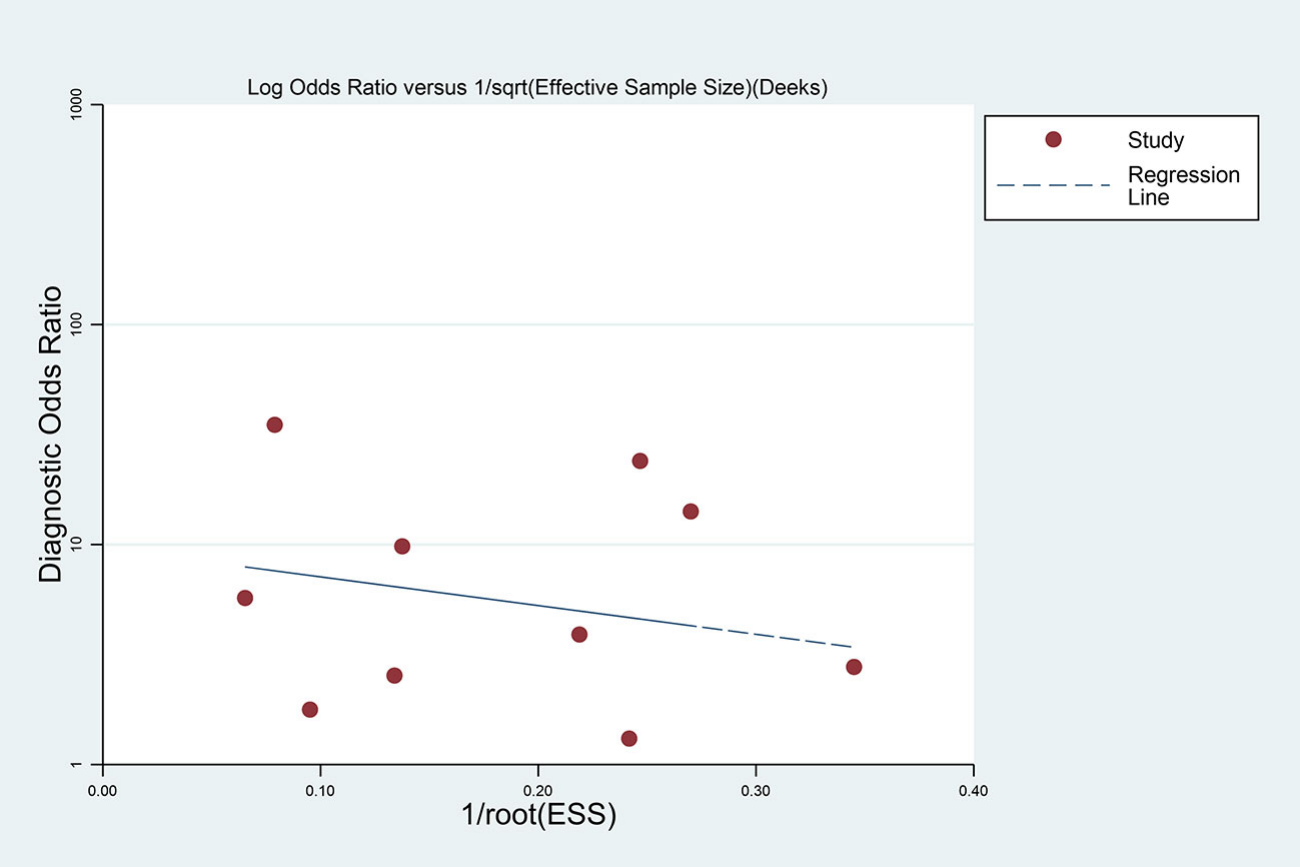
Funnel plot for publication bias. The horizontal axis is 1 divided by the square root of the effective sample size (ESS); values farther to the right indicate smaller sample sizes. The vertical axis shows the log diagnostic odds ratio (log DOR). Each dot represents one study; points are symmetrically scattered around the nearly flat regression line, with Deeks’ test p = 0.65 (>0.05), indicating no evidence of publication bias.

Publication bias was explored with a Deeks’ funnel plot and no significant differences were detected in this meta-analysis (p = 0.650) (**Figure 11**).

## 4 Discussion

Thyroid nodules are relatively common, but still 10%-15% of nodules are malignant (9). Ultrasound examination of the thyroid gland has been widely used for stratifying the risk of malignancy in thyroid nodules and aids in determining whether a FNA is necessary. When clinical indications are met, the preoperative application of FNA for the assessment of thyroid nodules can distinguish between benign and malignant conditions, reducing unnecessary surgical resection. This approach has been confirmed by a substantial body of literature. FNA cytology of thyroid nodules should be reported using the diagnostic categories outlined in the Bethesda System for Reporting Thyroid Cytopathology (10). Previously referred to as follicular neoplasm or suspicious for a follicular neoplasm (FN/SFN), Bethesda IV thyroid nodules have a 25–40% risk of malignancy (ROM) when considering NIFTP as malignant, but a 10–40% ROM when not considering NIFTP. According to the 2023 edition of the Bethesda reporting system, Bethesda IV thyroid nodules are classified as follicular neoplasm, with a 23–34% ROM when considering NIFTP and 0.2-30% when not considering NIFTP. Molecular testing and diagnostic gonadectomy are recommended as the management mode (11, 12). Although most Bethesda Category IV nodules are still benign tumors, a diagnostic lobectomy can offer a conclusive diagnosis. Patients may suffer unnecessary iatrogenic injury from lobectomies, particularly when the nodules have not produced clinical complaints. Thus, molecular testing would more safely direct clinical care if it could yield useful information regarding the type and risk of thyroid cancer (4).

Earlier studies show that *RAS* mutations are the most frequent alterations in thyroid FNA samples of indeterminate cytology. Among the three *RAS* subtypes—*NRAS*, *HRAS*, and *KRAS*—*NRAS* is mutated most often, followed by *HRAS*, with *KRAS* being the least frequently affected (13–16). The *RAS* gene encodes proteins that contribute to the *PI3K-AKT* and *MAPK* signaling pathways in cells, mainly by sending signals that encourage cell survival and proliferation. The *GTP*-binding domain (codons 12 and 13) or the *GTPase* domain (codon 61) often tightly controls the activation state of these proteins. Point mutations in the *RAS* gene, on the other hand, can result in the *RAS* protein becoming always active, which prevents apoptosis, encourages cell division, and may result in *DNA* damage and cell dedifferentiation. Activating mutations in the *RAS* gene are particularly important in thyroid cancer because they prevent apoptosis and encourage the transient growth of tumor epithelial cells, particularly when TSH (thyroid-stimulating hormone) is present. Through the *ERK* and *JNK* signal transduction pathways, this *RAS*-mediated cell proliferation, which is reliant on TSH, prevents programmed cell death. Furthermore, because elevated *RAS* expression suppresses the expression of thyroid-specific genes essential for preserving differentiation, activation of *RAS* can cause dose-dependent *DNA* damage and cell dedifferentiation. *RAS* mutations may contribute to the development of cancers as well as their progression. For instance, a greater risk of tumor metastasis is linked to mutations in *NRAS* exon2 (codon 61) (17). Furthermore, tumors with *RAS* mutations may change the immune cell composition in the tumor microenvironment, increasing the amount of M2-type tumor-associated macrophages and regulatory T cells (Tregs), which may encourage immune evasion and subsequent tumor progression. Thyroid cancer development, incidence, and resistance to treatment are thus significantly influenced by mutations in the *RAS* gene (17, 18).

According to our meta-analysis, *RAS* mutation showed a relatively low pooled sensitivity of 0.35 but a high specificity of 0.93 for distinguishing benign from malignant thyroid nodules in Bethesda category IV. This implies that there would be more false negatives and fewer false positives if *RAS* mutation were used as a diagnostic criterion. Therefore, in order to assist minimize overtreatment of thyroid nodules, conservative treatment rather than surgical gland resection is advocated for Bethesda Category IV thyroid nodules if *RAS* testing is negative and there are no evident clinical and imaging signs (19). To explore the sources of this heterogeneity, subgroup analyses were conducted with the following groupings: publication year (before and after 2016), detection of *RAS* typing (all detected/only 1–2 types detected), study type (prospective study/retrospective study), number of lesions (more than 42/less than or equal to 42), country (Asia and non-Asia), and research center (multicenter/single center). The results indicated that none of the subgroup analyses explained the heterogeneity. Subsequently, a sensitivity analysis was conducted by excluding studies one by one. It was found that only when Yuri E. et al. (8) was removed did the I^2^ value decrease significantly, from 58.5% to 3.4% (**Table 2**). This suggests that Yuri E. et al. (8) may have been a significant contributor to the heterogeneity observed in the meta-analysis, Yuri E. et al. (8) utilized Real-time LightCycler PCR and Fluorescence Melting Curve Analysis (FMCA) to detect *RAS* gene mutations. A novel method was developed to assess the proportion of epithelial cells in FNA samples by comparing the expression of the housekeeping gene GAPDH with the cytokeratin gene KRT7, which could lead to variations in results across studies. The sensitivity of Yuri E. et al. (8) is 0.54, and the specificity is 0.97, with the highest specificity among the ten included studies. This suggests that this method may be better for the detection of the *RAS* gene, thereby improving the diagnostic accuracy of the *RAS* gene for Bethesda Class IV nodules. Additionally, different detection techniques, such as Real-time Quantitative PCR, Pyrosequencing, Single Nucleotide Primer Extension, and High-Resolution Melting Curve Analysis, have varying sensitivities, specificities, and analytical throughputs, which may contribute to the variability in results. The collection, processing, and storage conditions of samples could affect the quality and quantity of *DNA* or *RNA*, thereby impacting the accuracy of mutation detection. Different studies may focus on detecting different *RAS* gene mutations (such as *NRAS*, *HRAS*, *KRAS*) or different mutation hotspots (such as codons 12, 13, 61, etc.). For instance, Wu (20) only tested *KRAS*, M. Decaussin-Petrucci (21) only tested *NRAS* and *HRAS*, and Luigi De Napoli (11) only tested *NRAS*, which could lead to heterogeneity in detection results. The threshold for determining mutation positivity may vary across studies, potentially affecting the sensitivity and specificity of mutation detection. Differences in data analysis methods and statistical processing may also impact the interpretation and comparison of results. Patient populations in different studies may differ in clinical and pathological characteristics, such as Cho (22) targeting *BRAF*-mutated patient population, which could influence the results of mutation detection and its correlation with clinical outcomes. The consistency and reproducibility of experimental results may be affected by different pathologists, laboratory conditions, and the experience and skills of technical staff. Patient populations from different regions may have genetic background differences, which could affect the frequency and spectrum of *RAS* gene mutations. William Clinkscales’ meta-analysis concludes (23): In many studies, the positive predictive value (PPV) for nodules with only *RAS* mutations is not high, and a conservative treatment approach is more recommended for low-risk malignancies.

**Table 2:** Results of sensitivity analysis.

No.	Author	Heterogeneity (%)
1	Yuri E. (8)	3.4
2	Sylvie Beaudenon-Huibregtse (24)	61.2
3	Aaron A. Stence (25)	58.1
4	Luigi De Napoli (11)	63.0
5	Rupendra T. Shrestha (26)	62.6
6	Hongxun Wu (20)	62.7
7	Jee Hyun An (16)	62.7
8	Markus Eszlinger (27)	49.7
9	M. Decaussin-Petrucci (21)	60.0
10	Yoon Young Cho (22)	62.3

The heterogeneity statistic for each row is the value recalculated after excluding that study.This suggests that Yuri E. et al. may have been a significant contributor to the heterogeneity observed in the meta-analysis.

This article uses the QUADAS-2 tool to assess the quality of the included literature. The quality assessment is conducted across four areas: patient selection, index test, reference standard, and flow and timing of patients. Overall, the literature included in this study is of high quality, and Deeks’ funnel plot indicates no significant publication bias. This meta-analysis was conducted strictly according to the PRISMA guidelines, making the pooled analysis results more reliable than the results of a single experimental study.

This study is innovative in a number of important ways. First off, our meta-analysis provides pooled values of sensitivity, specificity, and AUC, making it the first to our knowledge to specifically assess the diagnostic performance of *RAS* mutations in Bethesda Category IV thyroid nodules. Second, in cases where there is ultrasonographic suspicion of follicular neoplasm, we suggest an optimized clinical workflow that avoids repeat biopsies, improves diagnostic efficiency, and improves patient experience by performing molecular testing for *RAS* mutations concurrently with the initial FNA. These results show promise for wider clinical application and offer fresh evidence-based insights for the accurate treatment of ambiguous thyroid nodules.

Our study has several limitations (1): We only searched English-language databases, which may introduce language bias (2); The number of included studies was relatively small (i.e., 10 studies), and additionally, we were unable to obtain unpublished data (3); The included studies were not limited to any specific mutation detection methods, and these varying methodologies could also introduce confounding effects.

Pooling all subtypes, however, obscures the mutation’s true diagnostic yield for follicular variant of papillary thyroid carcinoma(FVPTC) and NIFTP because of its concentration in these entities. This evaluation might be improved by stratification by post-operative histology, but this analysis is not possible because the majority of publications only provide benign versus malignant outcomes. To determine the true effectiveness of *RAS* across various thyroid cancer subtypes, prospective multicenter datasets with comprehensive subtyping or IPD meta-analyses are required.

In conclusion, this meta-analysis shows that *RAS* mutations have a modest diagnostic value for Bethesda Category IV thyroid nodules. Conservative treatment is advised over surgical gland excision for Bethesda Category IV thyroid nodules if *RAS* testing is negative and there are no discernible clinical signs or imaging signs.

## Data Availability

The original contributions presented in the study are included in the article/supplementary material. Further inquiries can be directed to the corresponding authors.
